# Obesity as a clinical predictor for severe manifestation of dengue: a systematic review and meta-analysis

**DOI:** 10.1186/s12879-023-08481-9

**Published:** 2023-07-31

**Authors:** Chao-Ying Chen, Yu-Yao Chiu, Yu-Cheng Chen, Chung-Hao Huang, Wen-Hung Wang, Yen-Hsu Chen, Chun-Yu Lin

**Affiliations:** 1 Division of Infectious Diseases, Department of Internal Medicine, Kaohsiung Medical University Hospital, Kaohsiung Medical University, Kaohsiung, 807 Taiwan; 2 School of Medicine, Graduate Institute of Medicine, College of Medicine, Center for Tropical Medicine and Infectious Disease Research, Kaohsiung Medical University, Kaohsiung, 807 Taiwan; 3 Institute of Medical Science and Technology, School of Medicine, College of Medicine, National Sun Yat-Sen University, Kaohsiung, 804 Taiwan; 4 Department of Biological Science and Technology, College of Biological Science and Technology, National Yang Ming Chiao Tung University, HsinChu 100, Taiwan; 5 Department of Medical Biochemistry and Microbiology, Department of Surgical Sciences, Uppsala University, Uppsala, Sweden

**Keywords:** Dengue, Obesity, Prognosis

## Abstract

**Background:**

Severe dengue often leads to poor clinical outcomes and high mortality; as a result, it is of vital importance to find prognostic factors associated with the severe form of dengue. Obesity is known to deteriorate many infectious diseases due to impaired immune responses. Several studies have suggested that obese patients with dengue infection tend to have more severe manifestations with poorer prognosis. However, a firm conclusion could not be drawn due to the varied results of these studies. Here, we aimed to conduct a systematic review and meta-analysis to investigate the association between obesity and dengue severity.

**Methods:**

A literature search for relevant studies was conducted in PubMed, Embase, Ovid Medline and Cochrane from inception to September 9, 2022. The two main keywords were “dengue” and “obesity”. Mantel-Haenszel method and random effects model was used to analyze the pooled odds ratio with 95% confidence intervals.

**Results:**

A total of 15 article involving a total of 6,508 patients were included in the meta-analysis. Included patients in most studies were hospitalized pediatric patients. Only one study included adulthood data. Three cohort studies, four case-control studies, and one cross-sectional studies found a significant association between obesity and dengue severity. In contrast, three cohort studies, three case-control studies, and one cross-sectional study reported no significant relationship between obesity and dengue severity. Our analysis results showed that patient with obesity is 50% (OR = 1.50; 95%CI: 1.15–1.97) more likely to develop severe manifestation of dengue.

**Conclusion:**

This meta-analysis revealed that overweight could be a clinical predictor for severe disease for pediatric patients with dengue infection.

**Supplementary Information:**

The online version contains supplementary material available at 10.1186/s12879-023-08481-9.

## Background

Dengue fever is one of the most common arthropod-borne diseases around the globe [1, 2]. The disease is transmitted by vectors such as Aedes aegypti carrying dengue virus (DENV). It is estimated there are 390 million dengue infections around the world yearly [1]. In Taiwan, the burden of dengue was substantial, resulting in an annual loss of 115.3 disability-adjusted life-years (DALYs) per million population. The economic costs associated with dengue were primarily attributed to hospitalization expenses and the loss of productivity due to deaths occurring during epidemic years [3].

Most dengue-infected patients present with asymptomatic or inapparent infections[4]. According to previous literature, only a quarter of the dengue-infected patients will have primarily self-limiting symptoms [2]. The vexing part about a dengue endemic, however, is that a portion of the asymptomatic patients will develop severe dengue or even dengue shock as the disease progresses. Those with these conditions will have a horrendously high case fatality rate of 12–44% [5]. Luckily, with appropriate supportive management, such as intravenous fluid therapy, the mortality rate for dengue patients can be suppressed to less than 1%. Therefore, the importance to develop a clinical predictor for dengue patients during the time of an endemic is imperative [6, 7].

Overweight and morbid obesity (MO) are conditions considered to be prevalent among developed and developing countries. Although modern medicine has confirmed that obesity will have a negative impact on infectious diseases, such as coronavirus disease 2019 (COVID-19) [8], its role in dengue infection remains debated, possibly due to the heterogenicity of obesity definition [9, 10]. Previous related literature often based their data collected from different regions with different criteria for the overweight group and normal-weight group.

Obesity will elicit several negative physiological changes, including immune, respiratory, and circulatory systems. It is not only a risk factor for countless morbidities but also considered to be a negative prognostic factor for multiple infectious diseases [11–13]. The impaired immunity causes the obese patient to have a higher rate of post-surgery infection [14, 15] and a higher chance of getting severe viral infections like coxsackievirus, encephalomyocarditis virus, Influenza A, virus and SARS-CoV-2 [16–20].

Several biomarkers have been identified and proved effective in predicting the course of the disease in the past [21–23]. This systematic review and meta-analysis aimed to identify the relationship between dengue development and nutritional status, especially obesity, to investigate whether it can predict dengue prognosis.

## Methods

This systematic review and meta-analysis was conducted adhering to the principles described in the Cochrane Handbook [24] and the Preferred Reporting Items for Systematic Reviews and Meta-Analyses (PRISMA) 2020 statement [25]. As this study was a review of previously published studies, ethical approval or patient consent was waived.

### Information sources and search strategy

PubMed, Embase, Ovid Medline, and Cochrane were searched from inception to September 9, 2022, for relevant studies. We utilized the Patient, Investigated condition, Comparison condition, and Outcome (PICO) format to structure our review question: “In a patient with dengue, will the nutritional status become an effective clinical predictor for potential severe dengue?“ Accordingly, the patient population was defined as “patients infected with dengue.“ The investigated condition focused on “overweight patients,“ while the comparison condition involved “patients with healthy weights.“ Our primary outcome of interest was the “dengue prognosis.“

We used Boolean logic operator ‘AND’ to organize our two main keywords “dengue” and “obesity” into free text terms. Other synonyms were also included by using ‘OR’. A detailed list of free text terms of the search strategy is provided in **Additional file 1**. No filter or limit is used during the search. The search was complemented with hand searching of the reference lists of relevant studies.

### Eligibility criteria

This systematic review includes all observational and interventional studies that aim to compare the clinical development in dengue patients with different nutritional statuses, especially obesity. In vitro and animal studies, case reports, case series, reviews, letters, and conference abstracts were excluded.

### Outcome measure

In this study, we hope to assess whether obese patients tend to develop severe dengue. As a result, we focused on the one major clinical outcome of dengue infection that will differentiate dengue patient from mild infection: the development of severe manifestation of dengue. Due to the change in severity classification of dengue in 2009, we categorize the primary outcome according to the two different World Health Organization (WHO) criteria (Table 1).

**Table 1:** Outcome measurements of the present study. D, dengue; DF, dengue fever; WS, warning sign; SD, severe dengue; DHF, dengue hemorrhagic fever; WS, warning sign; DSS, dengue shock syndrome

Outcome	Development of severe manifestation of dengue	
Definition	Mild dengue infection	Severe manifestation of dengue infection
	DF, DHF I, II D with(out) WS	SD, DSS, DHF III, IV

### Collection process

Database search, title and abstract screening, full-text evaluation, data extraction, and meta-analysis were performed. Records were exported into EndNote 20 for duplicate removal, sorting, and further screening. The basic characteristics of the studies including author, year of the study and, article type were documented on a form using Microsoft Word 2019 to identify the studies. Inclusion criteria of the study population, dengue diagnostic criteria used and numbers of mild and severe dengue in patients with various weight categories were extracted as outcome. Missing data was either sought from other systematic review [26] or excluded from the study, no computed data was made.

### Statistical analysis

Review manager 5.4.1 was used to generate the forest plots, heterogeneity, and effect estimation using Odds Ratio (OR). Mantel-Haenszel method and random effects model was used. We considered p < 0.05 as a statistically significant differences while the confidence interval was set to be 95%.

### Bias assessment

Risk of bias assessment of the included records was evaluated using Newcastle-Ottawa Scale (NOS) while cross sectional study was evaluated by modified NOS suggested by Modesti et al. [27]. Standard NOS contains 3 domains and a total score of 9 (Table 2). A score of 7 or more defines a high quality records. A score of 5 or more defines a fair quality record while less defines a poor quality record [28, 29]. Publication bias was assessed by a funnel plot using Review manager.

**Table 2:** Quality rating in Newcastle-Ottawa Scale [29]

Quality rating	Selection Domain	Comparability Domain	Outcome Domain
Good	≥ 3	≥ 2	≥ 2
Fair	2	≥ 1	≥ 2
Poor	0 ~ 1	0	0 ~ 1

### Statistical analysis

We synthesized the data using the criteria of dengue severity mainly due to the massive heterogeneity in the definitions of overweight and obesity among all the included studies. We synthesized 15 records into the development of severe manifestation of dengue analysis. We obtained funnel plots to assess publication bias in the analysis.

A subgroup analysis was performed to reduce heterogeneity by annihilating potential bias that different study designs might cause; we divided our included study into three categories: cohort, case-control, and cross-sectional.

We also performed an additional analysis by combining data from multiple studies that used a common, yet different cutoff point.

## Results

### Study selection

**Fig. 1:**
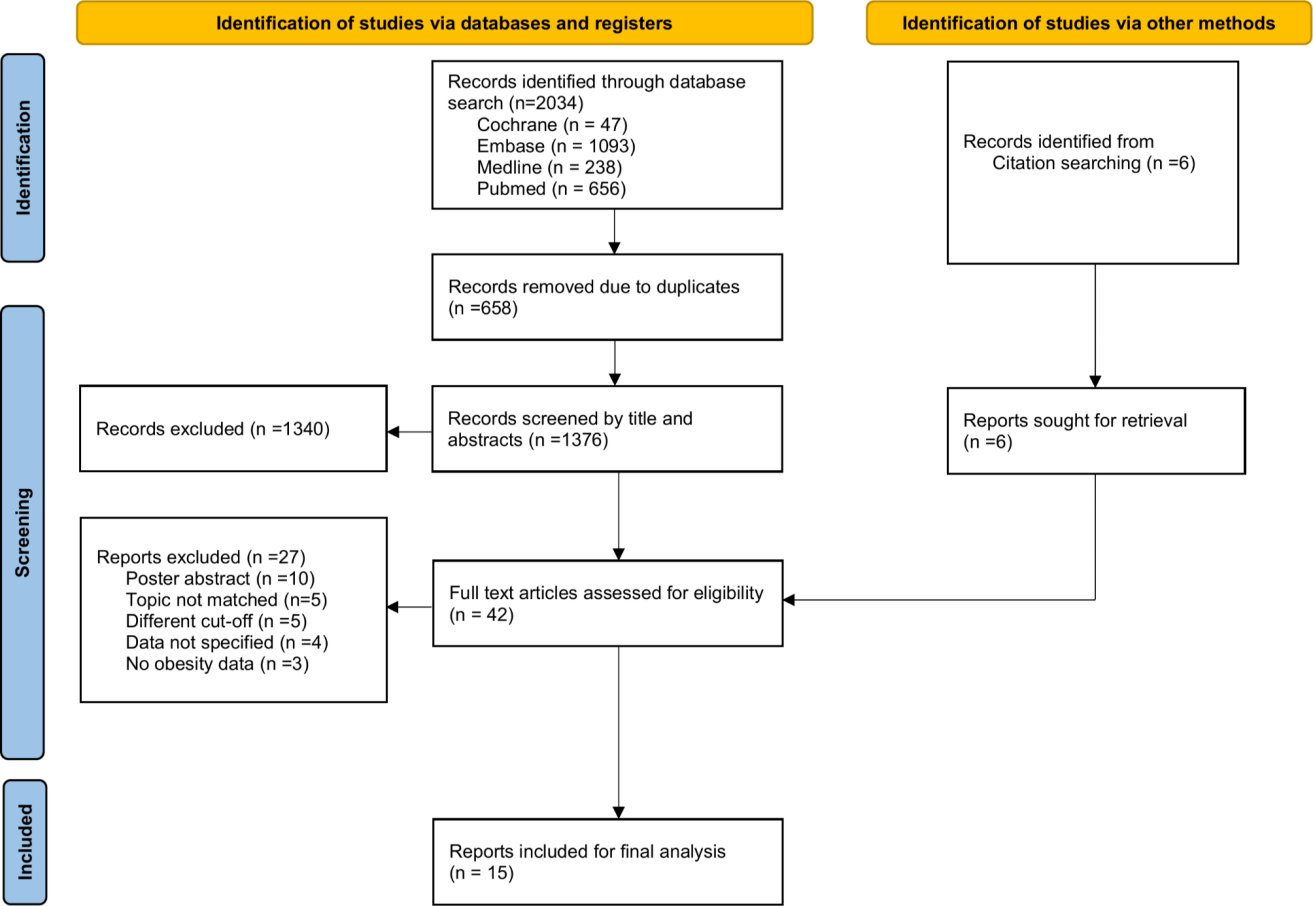
PRISMA 2020 flow chart of record acquisition

A total of 2,034 reports were identified from electronic database search and six records were found from two systematic reviews [26, 30]. Among them, 658 records were removed manually by authors and automatically by EndNote due to duplication. Out of the 1,376 records included for abstract screening, we excluded 1,340 records. With seven records identified from the citation search, we have 42 full texts retrieved in total to determine eligibility. In the 27 excluded records, we have 10 poster abstracts removed, five reports failed to match our outcome of interest, four records did not specify their crude data and three records focus on malnutrition patients and not specifying obesity data. We also excluded five studies that, while relevant to our desired outcome, utilized different cutoff points for defining dengue severity compared to the other included studies (Fig. 1).

### Characteristics of included studies

The main characteristics of included studies are summarized in Table 3. Of these fifteen studies, six were cohort studies (40.0%) [31–36], seven were case-control studies (46.7%) [37–43], and two were cross-sectional studies (13.3%) [44, 45]. A total of 6,508 patients were analyzed, with inclusion criteria starting from age 0 to the highest observed age of 73 year-old. The patients included in most studies were hospitalized pediatric patients. Only one study included adulthood data [36], and one study listed outpatient patients into population for analysis [45]. twelve studies applied the WHO 1997 classification for dengue severity. Included studies were conducted from 1995 to 2020 in endemic areas of Asia (Thailand, [32, 38, 40, 42, 45] Indonesia, [31, 33, 34, 37, 39, 41, 43, 44] Malaysia [36]) and Latin America (Paraguay [35]).

**Table 3:** Main characteristics of included studies

Reference	Study design	Country	Study period	xCriteria	Dengue classification		Age^a^	Sex, males	Variable and criteria of obesity	Associated outcomes	Confirmatory diagnosis
					Dengue, n (%)	Severe dengue, n (%)					
Chuansumrit, 2000	Cohort	Thailand	1 year (date not specified)	WHO 1986	15 (8.6%) DF; 116 (66.3%) DHF grade I and II	44 (25.1%) DSS (DHF grade III and IV)	Mean: 9y6m (range: 9 m–16y5m) [children]	51.4%	Variable: BW/age; obesity: BW/age > 75th percentile	Patients with BW/age > 50^th^ had a higher chance of DHF grade III or IV (P = 0.039)	Serological confirmation
Basuki, 2003	Cohort	Indonesia	September 2000 to September 2001	WHO 1997	4 (9.8%) DF; 7 (17.1%) DHF grade I, II	30 (73.2%) DHF grade III	81.7 ± 36.6 mo (range: 24–156 mo) [children]	46.3%	N/S	Higher proportion of obesity in DHF grade III than in DHF grade I (83.3% vs. 16.7%)	Serological confirmation (IgG and IgM)
Kan, 2004	Cohort	Indonesia	April to July 2000	WHO 1997	43 (51%) DHF without shock (DHF grade I and II)	42 (49%) DSS (DHF grade III and IV)	7.1 ± 2.88 y (range: 2.3–12.8 y) [children]	58.8%	Variable: BW/age according to WHO-NCHS standard; obesity: BW/age > 75th percentile	No significant difference in overweight between shock and non-shock (12% vs. 14%)	Serological testing; PCR
Kalayanarooj, 2005	Case-control	Thailand	1995 to 1999	WHO 1997	865 (19.1%) DF; 2544 (56.1%) DHF	1123 (24.8%) DSS	Case: 7.9 ± 3.8 y; control: 5.8 ± 3.5 y [children]	N/S	Variable: BW/age according to standard growth curve for Thai children; obesity: BW > 110% of ideal BW	Obese children had a higher risk of dengue infection (OR [95% CI] = 1.96 [1.55–2.5], P < 0.001)	Antibody test; PCR; virus isolation
Dewi, 2006	Cohort	Indonesia	January 1, 2003 to June 30, 2004	WHO 1997	42 (41.6%) DHF grade I, II, including 4 Overweight, 14 Normal ,24 Undernutrition	59 (58.4%) DSS, including 3 Overweight, 19 Normal ,37 Undernutrition	6.5 ± 3.6 y (range: 5 mo to 15 y) [children]	46.5%	N/S	No significant difference in nutritional status between shock and non-shock dengue (P = 0.57)	Serological confirmation (IgG and IgM)
Pichainarong, 2006	Case-control	Thailand	October 2002 to November 2003	WHO 1999 (Regional Guidelines)	105 (50%) Controls (DHF grade I and II)	105 (50%) Cases (DHF grade III and IV)	0–14 y [children]	N/S	Variable: BW/age and weight-for-height scales; obesity: ≥1.5 SD	Obese patients were at increased risk for more severe DHF (OR [95%CI] = 2.77 [1.19–6.45])	PCR
Junia, 2007	Case-control	Indonesia	January 2004 to December 2005	WHO 1997	400 (66.7%) Control (DHF)	200 (33.3%) Cases (DSS)	Case: 7.1 ± 3.2 y; control: 7.3 ± 3.5 y [children]	49.3%	Variable: Weight/height WHO-NCHS; overweight: 110–119%; obesity: >120%	Overweight was an independent risk factor for DSS (OR [95%CI] = 1.97 [1.29–3.08])	Serological confirmation (IgG and IgM)
Tantracheewathorn, 2007	Case-control	Thailand	January 2003 to December 2005	WHO 1997	110 (66.7%) Controls (DHF grade I, II)	55 (33.3%) Cases (DSS)	Total: 10.3 ± 3.3 y; case: 9.8 ± 3.5 y; control: 10.6 ± 3.2 y [children]	Total: 51.5%; control: 68.2%; case: 31.8%	Variable: BW/age according to the National Growth References for Thai children; obesity: >120%	Nutritional status was not statistically different between groups	Serological confirmation (IgM and IgG)
Widagdo, 2008	Cross-sectional	Indonesia	March to May 2005	WHO 1999	41 (91.1%) DHF grade I, II	4 (8.9%) DHF grade III, IV	75 ± 35 mo	33.3%	Variable: BMI/age; overnutrition: N/S	Nutritional levels for each of the four grades of DHF were not significantly different (P > 0.05)	Serological confirmation
Widiyati, 2013	Case-control	Indonesia	June 2008 to February 2011	WHO 1997	226 (66.1%) Controls (DHF grade I, II)	116 (33.9%) Cases (DSS)	< 18 y [children];	Cases: 47.4%; control: 54%	Variable: BMI/age; obesity: BMI/age > 2 SD	Obesity was not an independent risk factor for DSS (OR [95%CI] = 1.03 [0.32–3.31])	Serological confirmation (IgM and IgG)
Putra, 2014	Case-control	Indonesia	June 2011 to March 2012	WHO 1997	47 Controls (DF, DHF I and II)	47 Cases (DSS)	9 mo to 12 y [children] case: 75.5 ± 36.9 mo control: 88 ± 35.3 mo	Cases: 23%; controls: 49%	Variable: Body weight; obesity: N/S	No significant association between nutritional status and the severity of dengue (P = 0.268)	Serological confirmation (IgM and IgG)
Lovera, 2016	Cohort	Paraguay	2012–2013	WHO 2009	117 (24.8%) Without shock	354 (75.2%) Shock	10 ± 4 y [children]	51%	Variable: Body weight; obesity: N/S	Neither malnutrition, obesity nor overweight was associated with DSS (OR = 1.2, 95%CI = 0.7–2, P = 0.35)	Serological confirmation (IgM and IgG); immunochromatography assay for dengue NS1; PCR
Tan, 2018	Cohort	Malaysia	April- July 2015	WHO 2009	301 (89.9%) dengue with any warning sign	29 (8.7%) severe dengue	Median 30.2 y (range: 12.3–73.2 y) [adults, adolescents]	56.7%	Variable: BMI (for adults) and BMI/age (for adolescents [12–18 y]); obesity for adults: BMI ≥ 27.5 kg/m^2^; obesity for adolescents: BMI/age > 85% according to CDC growth chart	Obese patients my develop more severe forms of Dengue infection	NS1 antigen positive
Kurnia, 2019	Case-control	Indonesia	March to May 2019	WHO 1997	22 (50%) Controls (DHF grade I or II)	22 (50%) Cases (DHF grade III or IV)	11.1 ± 4.3 y (range: 3–17 y) [children]	63.6%	Variable: BMI/age obesity: BMI/age > 85% according to CDC growth chart	Obesity was significantly associated with DSS (OR [95%CI] = 7.734 [1.910-31.321], P = 0.004)	N/S
Te, 2022	Cross-sectional	Thailand	January 2017 to December 2019	WHO 1997 and WHO 2009	[WHO 1997] 296 DF, 48 DHF grade I and II; [WHO 2009] 279 Non-severe dengue	[WHO 1997] 11 DHF grade III and IV; [WHO 2009] 76 Severe dengue	Median (IQR) 15 (12–16) y	62.3%	Variable: BMI/age according to WHO growth chart; overweight: +2 SD; obesity: > +3 SD	Higher proportions of plasma leakage cases were overweight compared with those with mild plasma leakage (45.5% vs. 18.8%), but no difference in nutritional status was observed in patients with different dengue severity	Dengue NS1 test; serological confirmation (IgM)

^a^ mean ± SD unless otherwise indicated. *Malavige 2006 was not included in neither of our analysis due to cut-off point difference

Abbreviations: DF, dengue fever; DHF, dengue hemorrhagic fever; DSS: dengue shock syndrome; BW/age, body weight-for-age; BMI, body mass index; BMI/age: BMI-for-age; BAZ, BMI-for-age Z-score; HAZ, height-for-age Z scores; WAZ, weight-for-age Z scores; WHZ, HAZ and weight-for-height Z scores; rRT-PCR, real-time reverse transcriptase-polymerase chain reaction; N/S, unspecified or unavailable

The overweight or obese status was defined using body weight or body weight-for-age in five studies [32, 34, 38, 40, 42]; body mass index (BMI) or BMI-for-age in four studies [36, 39, 43, 45] and weight/height% in one study [37]. Other five studies did not specify the criteria for obesity [31, 33, 35, 41, 44]. Confirmatory diagnosis of dengue was performed by laboratory tests, such as serology for anti-dengue IgM and/or IgG (12/15, 80.0%) [31–35, 37, 38, 41–45] dengue nonstructural protein 1 (NS1) antigen test (3/15, 20.0%) [35, 36, 45], identification of viral ribonucleic acid (RNA) using polymerase chain reaction (PCR) (4/15, 26.7%) [34, 35, 38, 40], and isolation of viruses (1/15, 6.7%) [38]. The majority of studies employed multiple diagnostic assays to confirm the diagnosis of dengue, except for one study that did not specify the method used [39]. Warning signs or symptoms were reported as follows: skin bleeding (petechiae, purpura, hematoma) (9/15, 60.0%) [31–33, 35, 37, 38, 41, 42, 44], epistaxis (4/15, 26.7%) [31–33, 41], hematemesis (3/15, 20.0%) [31–33], melena (3/15, 20.0%) [31–33], hepatomegaly (7/15, 46.7%) [33, 34, 37, 38, 41, 42, 44], abdominal pain (8/15, 53.4%) [33–38, 41, 42], vomiting and nausea (8/15, 53.4%) [33–38, 41, 42], diarrhea (4/15, 26.7%) [33, 36, 42, 44], thrombocytopenia (4/15, 26.7%) [33, 36, 37, 44], seizure (1/15, 6.7%) [33], bleeding gums (1/15, 6.7%) [32], pleural effusion (3/15, 20.0%) [33, 42, 44], ascites (1/15, 6.7%) [42], menorrhagia (1/15, 6.7%) [32] shock (1/15, 6.7%) [33], hemoconcentration (1/15, 6.7%) [33] and altered consciousness (1/15, 6.7%) [33].

Most of the studies classify dengue patients using the 1997 criteria of dengue fever (DF), dengue hemorrhagic fever (DHF), and dengue shock syndrome (DSS). Except two studies using 2009 WHO criteria [35, 36] and one study uses 1986 WHO criteria [32]. Most of the population included are diagnosed with DHF across all four stages.

### Obesity as a clinical predictor of development of severe manifestation of dengue

**Fig. 2:**
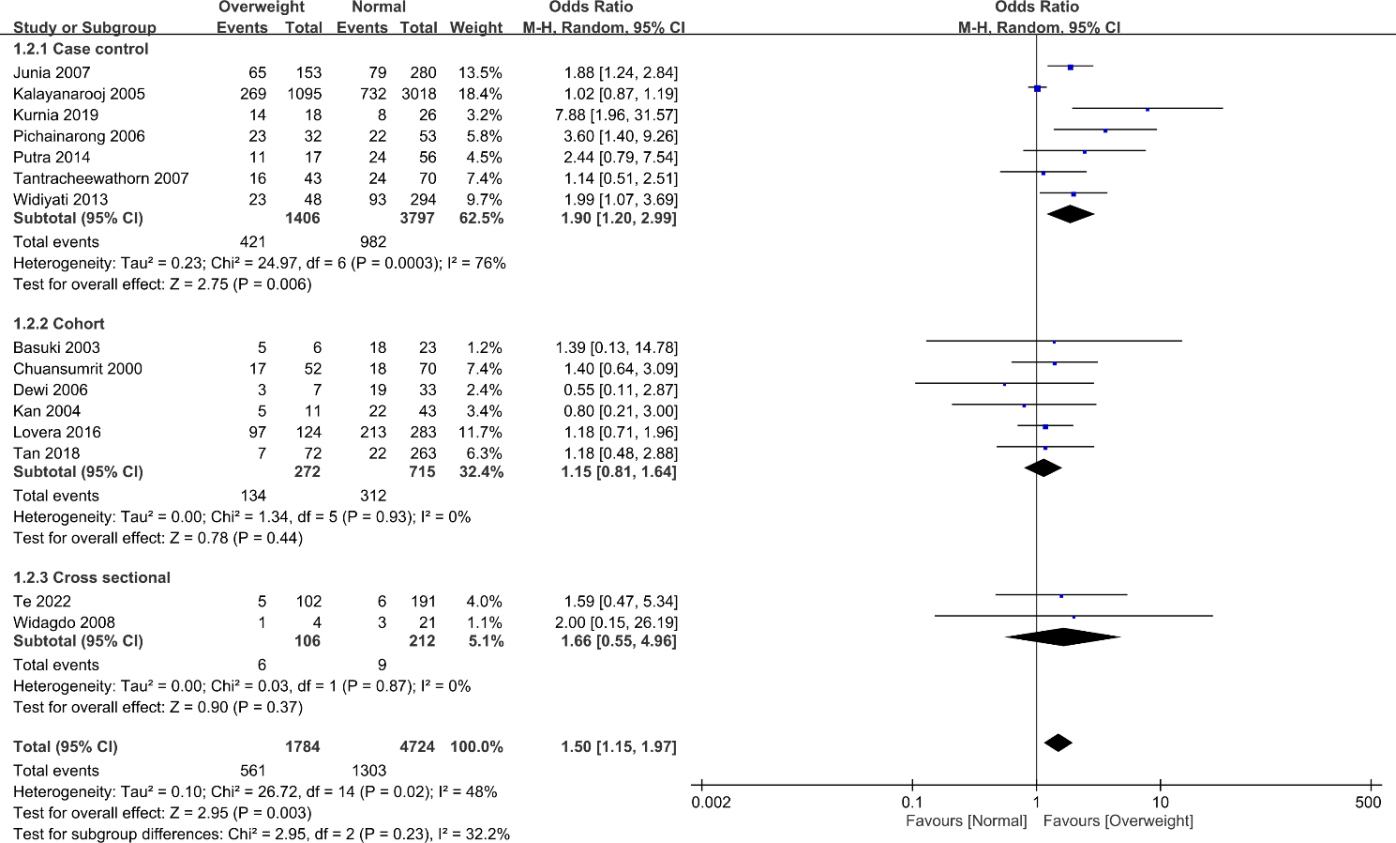
Development of DSS/SD comparison between over- and normal-weight patients

Overweight patients are 50% (OR 1.50, 95% CI 1.15–1.97) more likely to develop severe manifestation of dengue when compared to patients with normal weights. (Fig. 2). The result is statistically significant but with high heterogenicity (I^2^ = 48%, p = 0.02). In the subgroup analysis, only the studies with case-control design achieved statistical significance (OR 1.90, 95% CI 1.20–2.99) but even with a higher heterogenicity (I^2^ = 76%, p = 0.0003). The other two subgroups failed to demonstrate statistical significance but with lower heterogenicity.

**Fig. 3:**
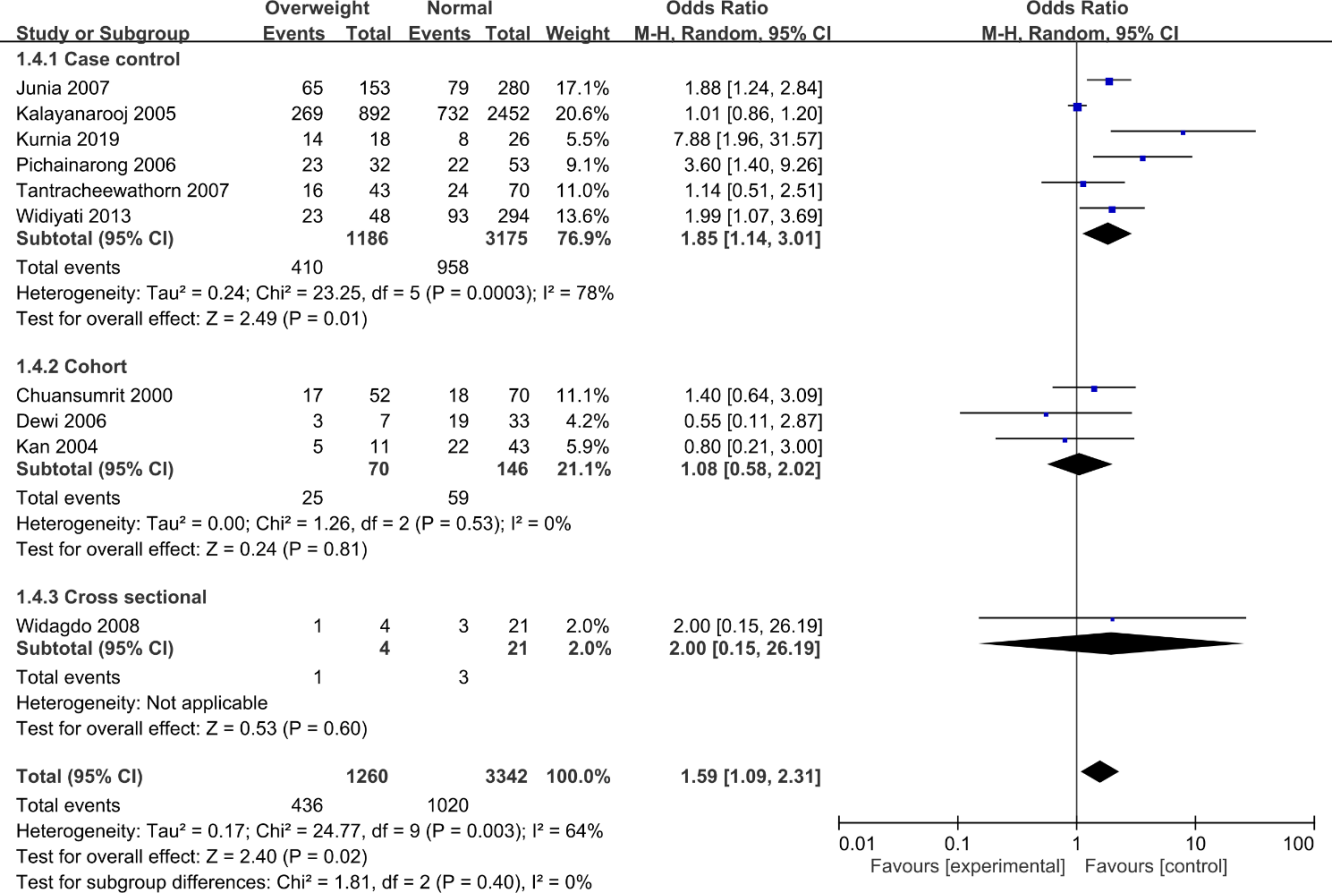
Events of DHF and DSS comparison between over- and normal-weight patients

We conducted a second analysis that including only hospitalized pediatric patients diagnosed with DHF by excluding records that included population diagnosed with DF (Fig. 3). However, we observed a finding similar to not excluding patients diagnosed with DF. In pediatric hospitalized patients diagnosed with DHF, overweight children are 59% (OR 1.59, 95% CI 1.09–2.31) more likely to develop shock signs. However, this result has an even higher heterogenicity (I^2^ = 64%, p = 0.003). In the subgroup analysis, only the studies with case-control design demonstrated statistical significance (OR 1.85, 95% CI 1.14–3.01) and a higher heterogenicity (I^2^ = 78%, p = 0.0003).

We conducted an additional analysis and found obesity patients have a statistically significant 16% increase in the development of dengue with concerning conditions. The concerning conditions are defined by patient with a final diagnosis across all four stages of DHF, dengue with warning signs and severe dengue. Further details about this analysis can be found in the supplementary materials (**Additional file 2**) accompanying our report.

### Risk of bias assessment

**Fig. 4:**
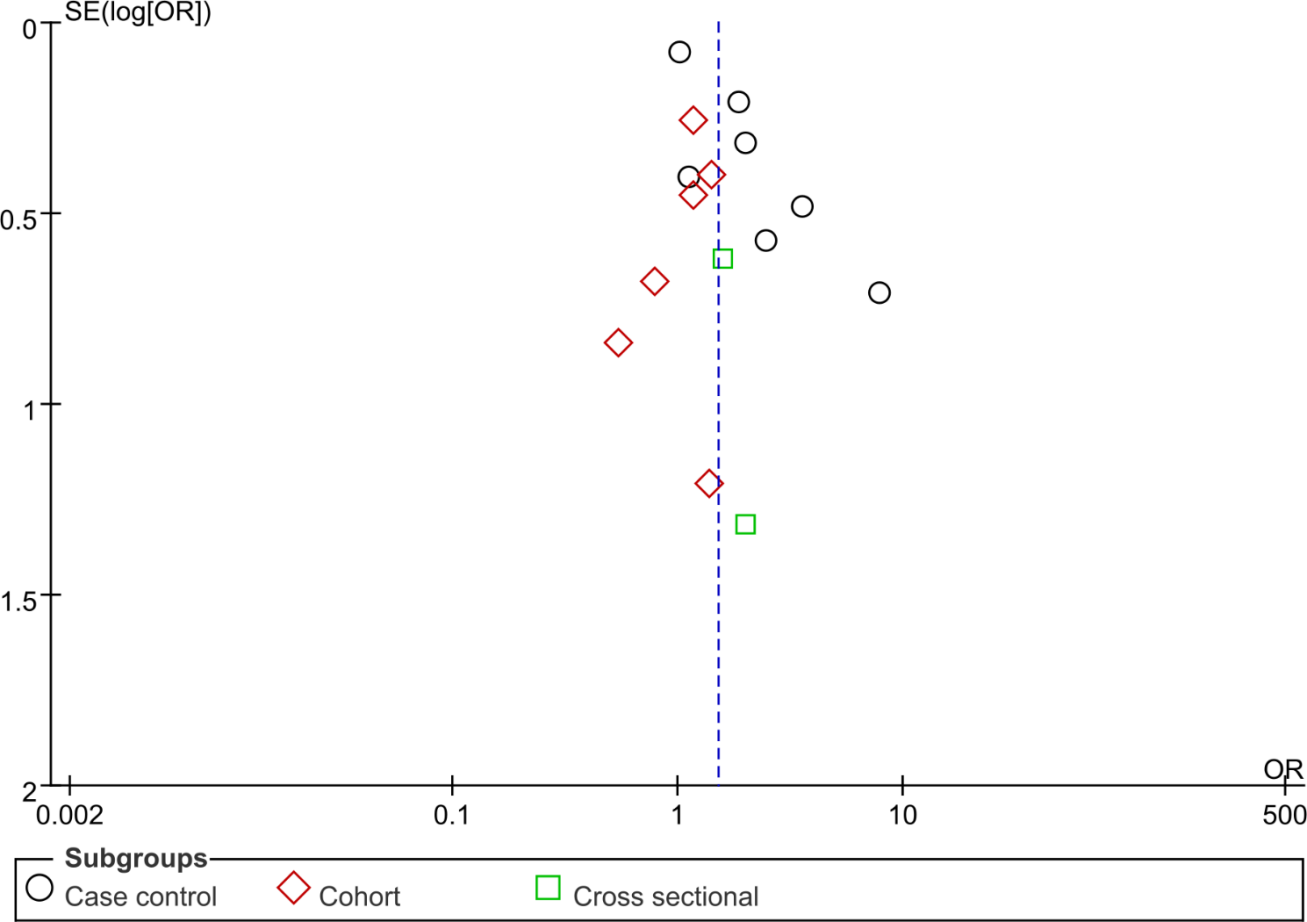
Funnel plot of Fig. 2 Development of DSS/SD comparison

**Fig. 5:**
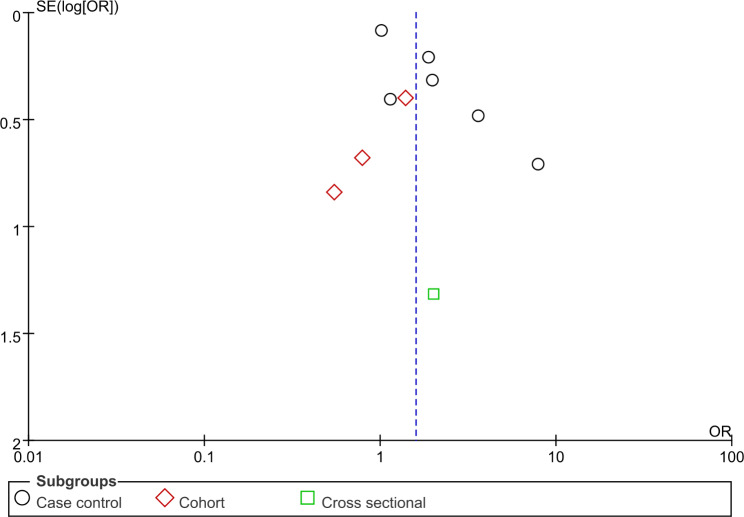
Funnel plot of Fig. 3 Events of DHF and DSS comparison

Publication bias of the analysis was not significant since we found both funnel plots represent approximate symmetrical shapes (Fig. 4 and Fig. 5).

Out of the 15 of studies evaluated, nine were classified as high-quality studies, while six were determined to be of fair quality.(Table 4) In terms of case-control studies, most of them scored 3 stars in the selection category [37–40, 42, 43], as they predominantly employed hospitalized patients as control. Majority of the studies did not receive any stars in the comparability category, indicating a lack of controlled factors. However, two studies did address variables related to obesity, such as comorbidity, age[36, 46], and dengue virus types [40], which contributed to enhancing the comparability of their findings. It is worth noticing that cross-sectional studies have to use modified NOS with a total score of 10. For a more detailed breakdown of the scoring, please refer to Additional file 3.

**Table 4:** Results of Newcastle Ottawa-score

Reference	Quality scores			
	Selection	Comparability	Exposure	Total
Chuansumrit, 2000	4	0	3	7
Basuki, 2003	4	0	3	7
Kan, 2004	4	0	3	7
Kalayanarooj, 2005	3	0	3	6
Dewi, 2006	4	0	3	7
Pichainarong, 2006	3	1	3	7
Junia, 2007	3	0	3	6
Tantracheewathorn, 2007	3	0	3	6
Widagdo, 2008	5	0	3	8
Widiyati, 2013	3	0	3	6
Putra, 2014	3	0	3	6
Lovera, 2016	4	0	3	7
Tan, 2018	4	1	3	8
Kurnia, 2019	2	0	3	5
Te, 2022	4	0	3	7

## Discussion

In this systematic review and meta-analysis, we found that obese patients were more likely to develop severe manifestation of dengue as manifested by dengue shock syndrome and severe dengue as compared with normal-weight patients. We showed that obesity is significantly associated with dengue severity. Thus, obesity could be a potential clinical predictor for severe dengue.

**Fig. 6:**
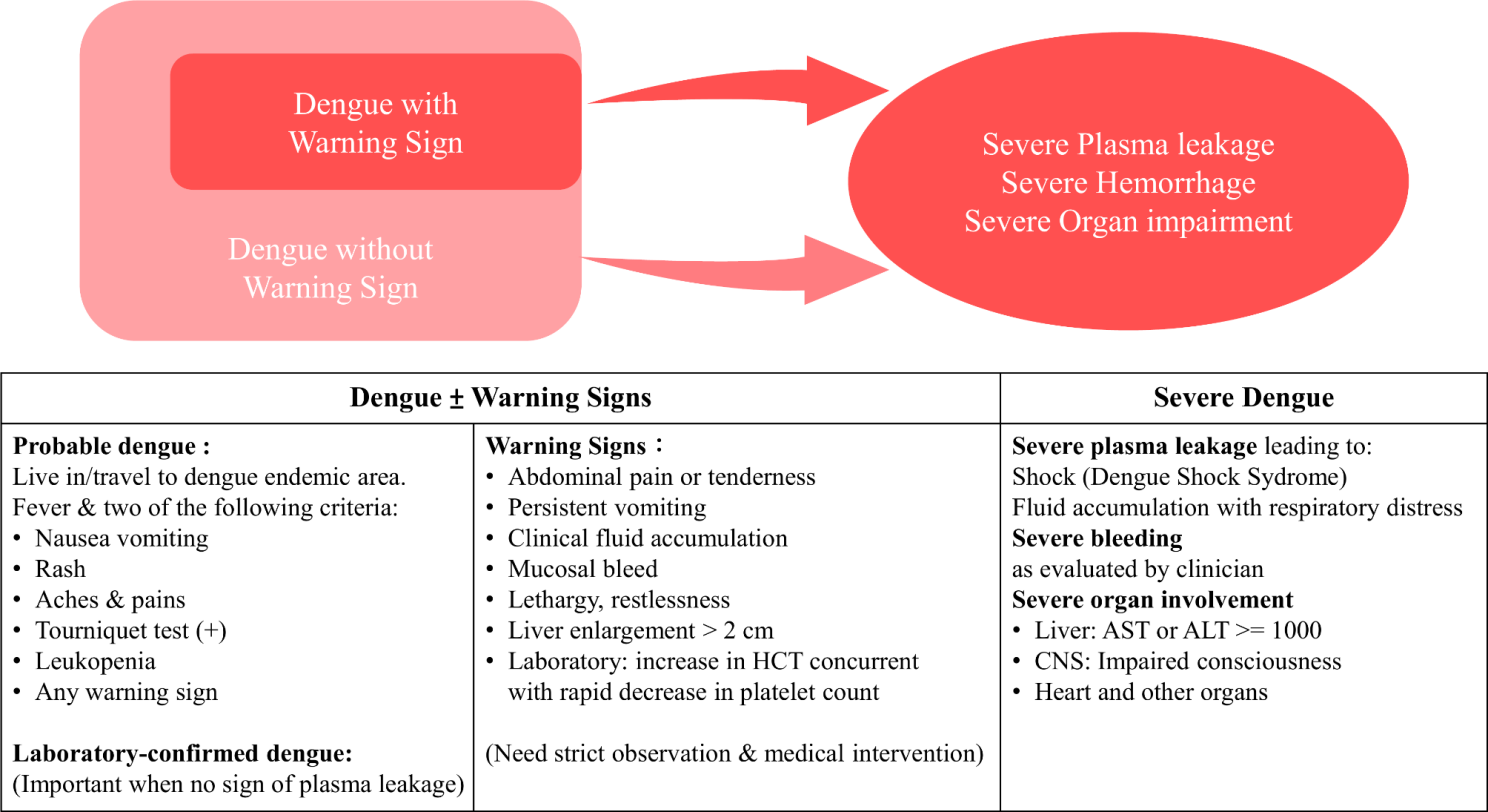
Suggested dengue case classification and levels of severity (adopted from WHO [4])

The heterogenicity in the dengue-type classification caused difficulties in defining a patient’s clinical conditions. In 1997 [47], WHO published a standard to classify dengue infection severity. In this standard, dengue disease was divided into three types based on the clinical presentations: mild DF, which is associated with self-limited disease; DHF, associated with vascular alterations including thrombocytopenia and granulocytopenia, as well as hepatomegaly; and DSS if systemic plasma leakage occurs. Yet, this classification method was considered to be underestimating the clinical burden of dengue fever due to the fact that some of the severe dengue patients did not match the clinical criteria of plasma leakage [48, 49]. Those patients who did not show significant bleeding did not receive the appropriate medical attention they needed [50, 51]. In 2009 [4], WHO revised the dengue severity level standard and thus regrouped the patients into Dengue and Severe dengue. In the patients classified as dengue, WHO further defines some worrying clinical presentations as warning signs. Those patients who present with warning signs are more likely to develop severe dengue and thus need more intensive care. In this study, we tried to eliminate this heterogenicity by designing a primary outcome focused on the presence of plasma leakage or shock (Fig. 6). Patients with plasma leakage are more likely to develop severe dengue and those who are in shock are in an abysmal condition.

Obesity is a risk factor for various infectious diseases [13]. Obesity-related susceptibility to infectious diseases is believed to be associated with an impairment of both innate and adaptive immune responses and vitamin D deficiency [8]. However, whether obesity is a risk factor or prognostic factor for severe dengue remains elusive. In a meta-analysis study by Tsheten et al., [52] the authors declared that obesity was not significantly associated with severe dengue disease. Nevertheless, in another meta-analysis study by Lima et al. [53], the authors showed that circulating total-cholesterol and low-density lipoprotein (LDL)‐cholesterol levels were inversely and significantly correlated with dengue severity, and suggested that the two factors can serve as routine markers for dengue severity. Indeed, nutritional status may post a significant impact on dengue infection. In an animal experiment, obesity will alter the cytokine change in dengue-infected mice. They also suffered from weight loss and thrombocytopenia compared to mice with a healthy weight [54]. In patients with dengue fever, obese patients tend to have comorbid acute kidney injury [24, 55].

According to Gallagher et al., [56] there are four main key mechanisms on how obesity may affect dengue infection. First, obesity will cause downregulation of AMP-Protein Kinase (AMPK) and thus buildup of lipids at the endoplasmic reticulum that favors viral replication. Second, an increase in adipokines production will lead to chronic inflammation, causing the C-reactive protein (CRP) elevation and imbalance of pro- and anti-inflammatory cytokines. They will further exacerbate the development of plasma leakage through dysfunction of endothelial and platelet. Third, adipokines can trigger downregulation of endothelial nitric oxide synthase (eNOS) and thus accrue the production of reactive oxygen species (ROS), which leads to the damage of the endothelial glycocalyx. Finally, the immunomodulation effect of obesity itself attenuates natural killer cells, B cells, and T cells responses to infection, boosting the inclination toward stronger cytokine pro-inflammatory response.

Compared to previous literature [26, 30], we have added three new articles [36, 39, 45] after 2018 into our analysis. We also attempted to reduce the heterogeneity of the definition of the population through measures like synthesis analysis pool based on population characteristics. We also intentionally excluded the malnourished population in one study [38] due to the ambiguous effects of malnutrition status on dengue infection.

### Limitation

This review has several limitations. First, most of the included population are hospitalized patients, which might have a different clinical care status and disease course from patients in the outpatient department. Considering that a majority of dengue patients presented with mild symptoms and did not require hospitalization, the results should be interpreted with caution. Second, the heterogeneity in this study is high. This may be due to the massive differences in classifying a patient as overweight from study to study. Due to the lack of original data, we did not make any adjustments or correlations to address this heterogenicity. It is also worth noting that in some included studies, the specific definition of obesity was not provided. Third, multiple factors, such as previous dengue infection, co-morbidities, or socioeconomic status might also affect a patient clinical status. According to Zulkipli et al. [26] obesity is associated with higher socioeconomic status, which may alter the medical-seeking behavior of individual patients. We did not take any measures to address this issue, and it should be taken into consideration. Lastly, this analysis is based on the final clinical diagnosis given by included data and may not be a complete presentation of the general patient population. Other factors such as end organ (s) failure, respiratory distress, co-morbidity, co-infections, and various other causes might contribute to the final presentation of the patient.

## Conclusions

In this study, we demonstrated that obesity can serve as a clinical predictor for patients with an unfavorable outcome of dengue infection. We found 50% more likely the development into severe manifestation of dengue infection for the overweight patients diagnosed with dengue. However, this result should be implemented into clinical practice with great caution since the heterogenicity of this study is high and the study population is limited.

## Electronic supplementary material

Below is the link to the electronic supplementary material.

Supplementary Material 1

Supplementary Material 2

Supplementary Material 3

Supplementary Material 4

Supplementary Material 5

## Data Availability

All data generated or analysed during this study are included in this published article.

## List of abbreviations

AMPK: AMP-Protein Kinase
BMI: body mass index
COVID-19: coronavirus disease 2019
CRP: C-reactive protein
DENV: dengue virus
DF: dengue fever
DHF: dengue hemorrhagic fever
DSS: dengue shock syndrome
LDL: low-density lipoprotein
NS1: nonstructural protein 1
eNOS: endothelial nitric oxide synthase
MO: morbid obesity
NOS: Newcastle-Ottawa Scale
PCR: polymerase chain reaction
PRISMA: Preferred Reporting Items for Systematic Reviews and Meta-Analyses
ROS: reactive oxygen species
RNA: ribonucleic acid
WHO: World Health Organization
