# Supplementary figures and images for: Obesity as a clinical predictor for severe manifestation of dengue: a systematic review and meta-analysis
Source: BMC Infect Dis. 2023 Jul 31;23:502. doi: 10.1186/s12879-023-08481-9 (PMC10388491; doi:10.1186/s12879-023-08481-9)

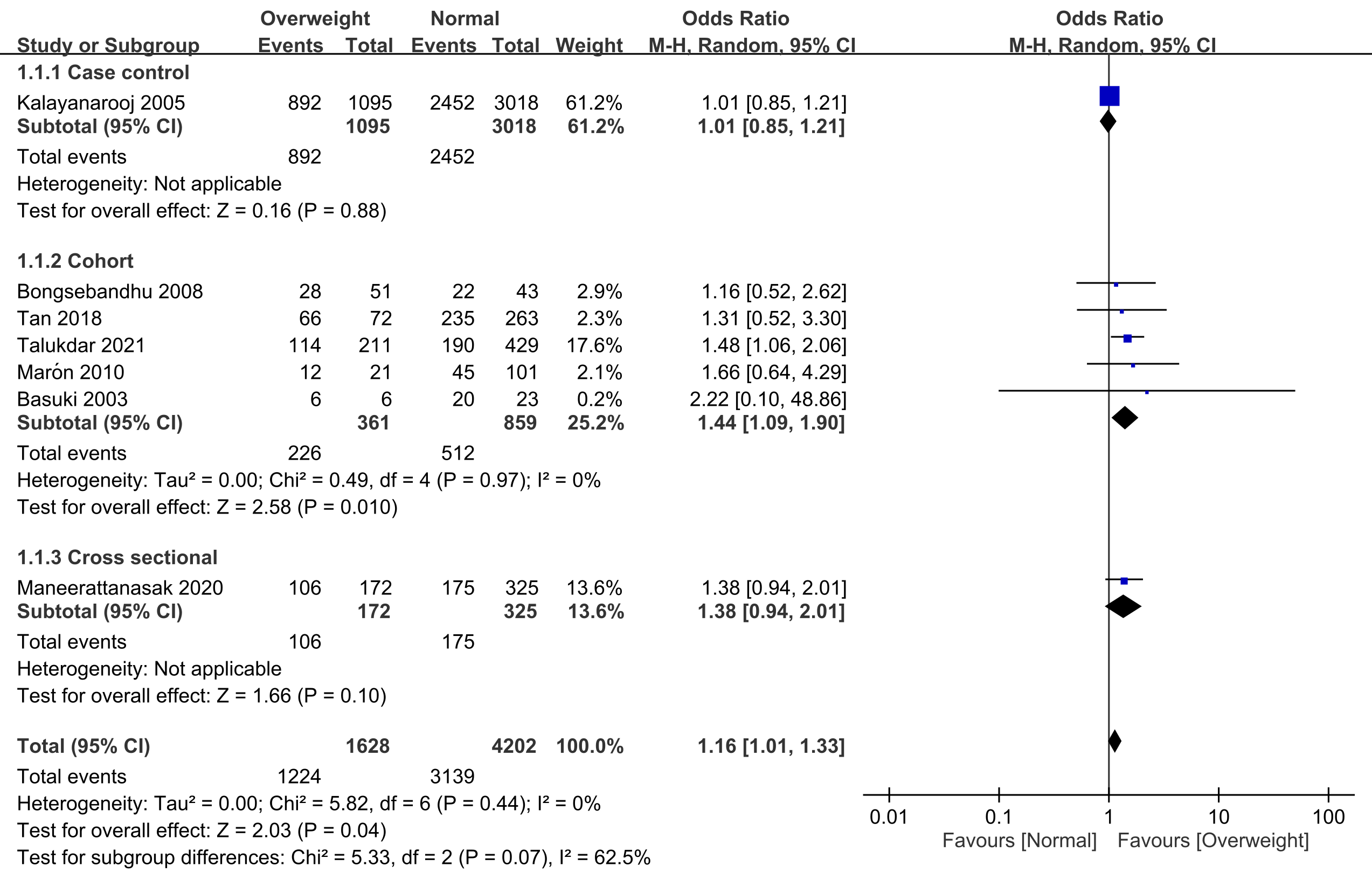

Supplement: Supplementary file 1 — Supplementary Material 1 [file 12879_2023_8481_MOESM1_ESM.png]

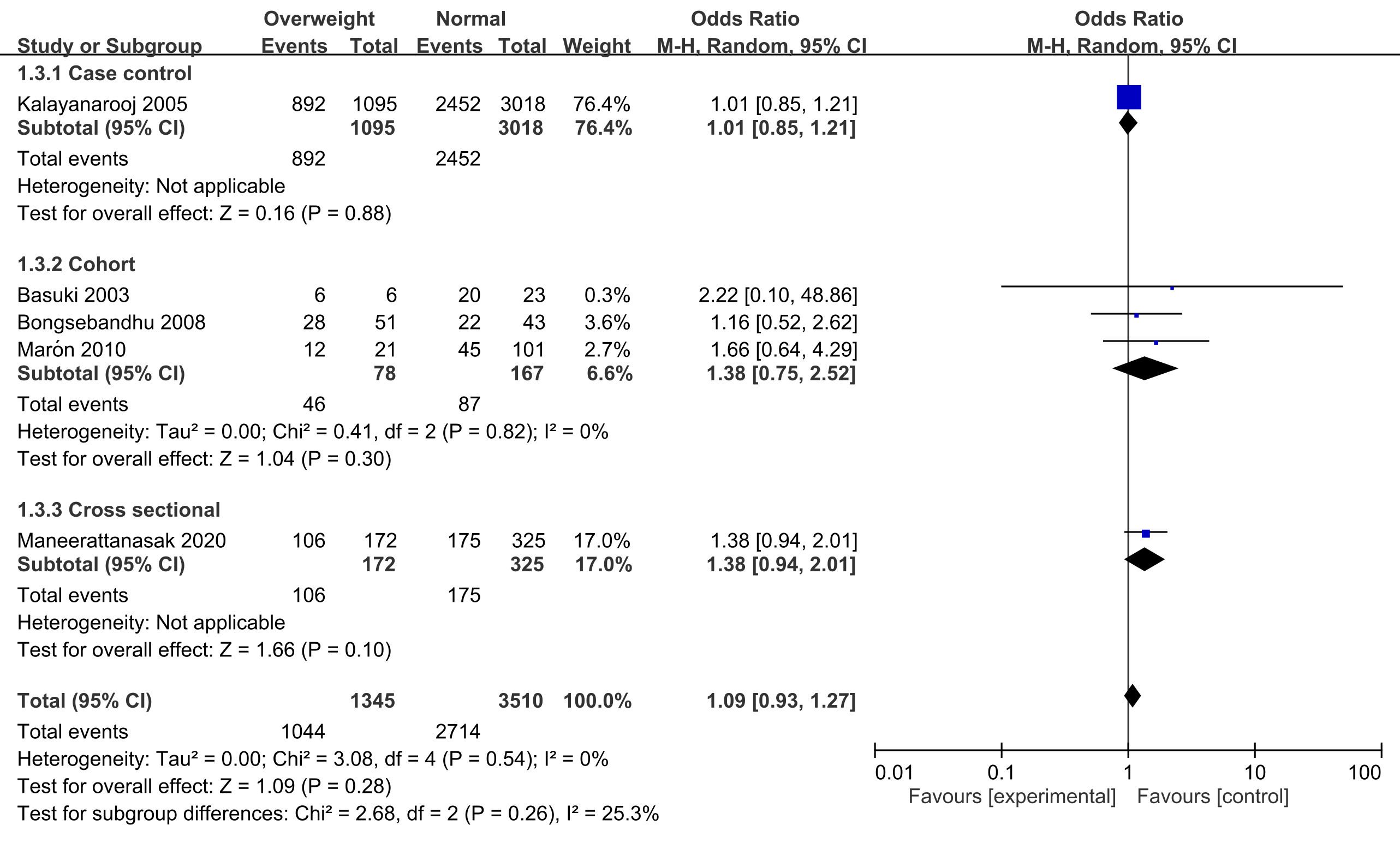

Supplement: Supplementary file 2 — Supplementary Material 2 [file 12879_2023_8481_MOESM2_ESM.png]
