# Supplementary material for: Obesity as a clinical predictor for severe manifestation of dengue: a systematic review and meta-analysis
Source: BMC Infect Dis. 2023 Jul 31;23:502. doi: 10.1186/s12879-023-08481-9 (PMC10388491; doi:10.1186/s12879-023-08481-9)
Supplement: Supplementary file 3 — Supplementary Material 3 [file 12879_2023_8481_MOESM3_ESM.docx]

**Table S1.** Text term used for initial database search

| ***Name of database*** | ***Search strategy (Free text term)*** |
| --- | --- |
| PubMed | (dengue) AND ((obes*) OR (adipo*) OR (-weight) OR (waist circumference) OR (bmi) OR (Body Mass Index) OR (nutriti*)) |
| Cochrane | #1(dengue)  #2(adipos*)  #3(obes*)  #4(nutriti*)  #5(body mass index)  #6(bmi)  #7(waist circumference)  #8(*weight)  #9(#2 OR #3 OR #4 OR #5 OR #6 OR #7 OR #8)  #10(#1 AND #9) |
| EMBASE | #10. (#1 AND #9)  #9. (#2 OR #3 OR #4 OR #5 OR #6 OR #7 OR #8)  #8. (-weight)  #7. ('waist circumference')  #6. (bmi)  #5. ('body mass index')  #4. (nutriti*)  #3. (obes*)  #2. (adipo*)  #1. ('dengue'/exp OR dengue) |
| Ovid Medline | 1 (Dengue/ or Severe Dengue/ or Dengue Virus/)  2 (adipo*.mp.)  3 (Obesity, Morbid/ or Obesity/ or obes*.mp.)  4 (Nutritional Status/ or nutriti*.mp. or Malnutrition/)  5 (Body Mass Index/)  6 (Obesity, Morbid/ or bmi.mp. or Pediatric Obesity/)  7 (Waist Circumference/)  8 (-weight.mp. or "Weights and Measures"/)  9 (2 or 3 or 4 or 5 or 6 or 7 or 8)  10 (1 and 9) |
