# Supplementary material for: Obesity as a clinical predictor for severe manifestation of dengue: a systematic review and meta-analysis
Source: BMC Infect Dis. 2023 Jul 31;23:502. doi: 10.1186/s12879-023-08481-9 (PMC10388491; doi:10.1186/s12879-023-08481-9)
Supplement: Supplementary file 4 — Supplementary Material 4 [file 12879_2023_8481_MOESM4_ESM.docx]

**Obesity as a clinical predictor of dengue with concerning conditions**

**Table S2.** Outcome measurements of the present study. D, dengue; DF, dengue fever; WS, warning sign; SD, severe dengue; DHF, dengue hemorrhagic fever; WS, warning sign; DSS, dengue shock syndrome.

| Outcome | Development of dengue with concerning conditions | |
| --- | --- | --- |
| Definition | Dengue without concerning conditions | Dengue with concerning conditions |
|  | DF  D without WS | SD, DHF  D with WS |

Four records[1-4] have applied a different cut-off point than all other studies and thus cannot be synthesized into our study. An additional analysis was performed using the different categorization to synthesize these results: Development of dengue with concerning conditions. (**Table S2**)

In our additional analysis, we find overweight patient is 16% (OR 1.16, 95% CI 1.01–1.33) more likely to develop dengue with concerning conditions **(Fig. S1)**. This result is statistical significance (p = 0.04) but possesses a relatively high heterogenicity among subgroups. (I^2^ = 62.5%, p = 0.07) In the subgroup analysis, the overweight patient is 44% (OR 1.44, 95% CI 1.09–1.90) more likely to have plasma leakage signs in the cohort group. We did not identify similar findings in the other two study-type subgroups, which only consist of one study.

Aiming to reduce heterogeneity in included population and case definition, we perform a second analysis that exclude adulthood data [5] and a record with self-defined criteria of plasma leakage[3], leaving hospitalize children included in the analysis **(Fig. S2)**. In the second analysis, we fail to demonstrate any differences in the events of two diagnoses between overweight and healthy children (OR 1.09, 95% CI 0.93–1.27) with a comparable lower subgroup heterogenicity. (I2 = 25.3%, p = 0.26) All subgroup analyses in the second analysis failed to demonstrate any significant findings.

| 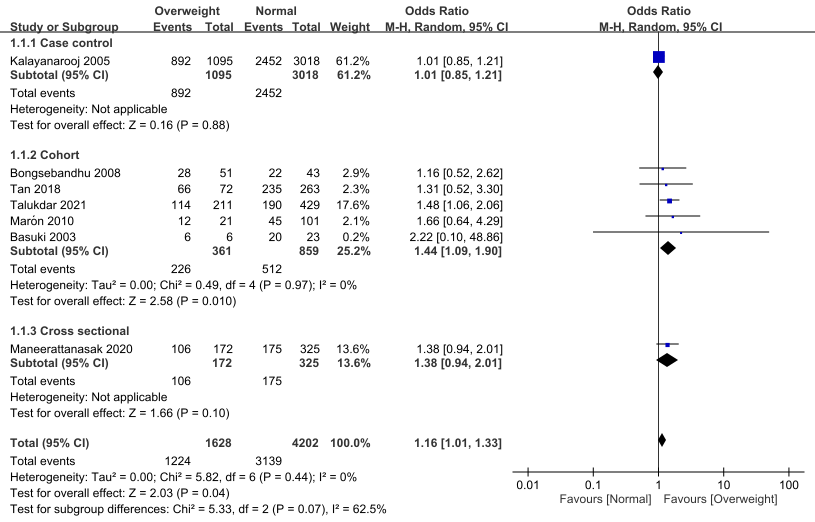 |
| --- |
| **Fig. S1**. Forest plots of random-effects meta-analysis for development of dengue with concerning conditions |

| 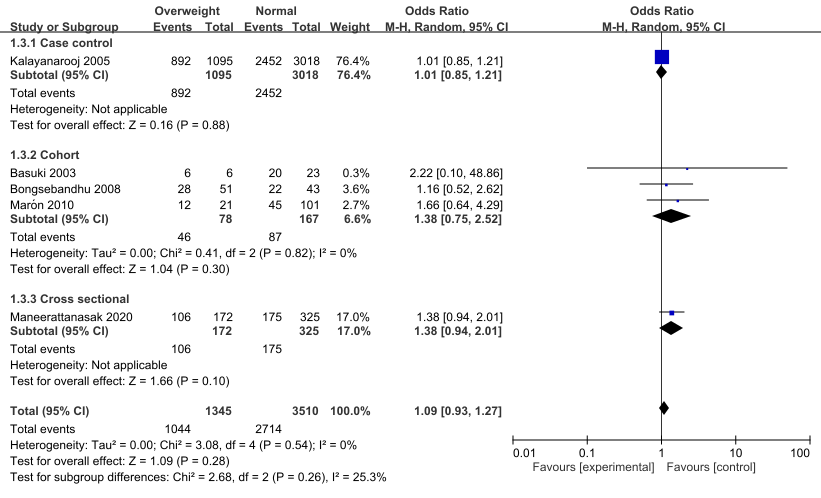 |
| --- |
| **Fig. S2**. Forest plots of random-effects meta-analysis for development of dengue with concerning conditions excluding two records that may contribute to heterogeneity |

We observed that the first result aligns with our analysis of the development of severe manifestations of dengue, as described in the manuscript. On the other hand, the second analysis, which had a smaller sample size, did not yield a statistically significant result. It is important to note that the grouping of dengue with concerning conditions includes patients who may have exhibited concerning conditions but did not experience a worsened clinical outcome.

Table S3 presents the four new studies that were included in our second analysis, all of which were of good quality. [1-4] **(Table S3)** Additional baseline characteristics can be found in **Table S4** . We were unable to obtain a funnel plot as there were less than 10 studies included in our analysis.

**Table S3.** Results of Newcastle Ottawa-score.

| **Reference** | **Quality scores** | | | |
| --- | --- | --- | --- | --- |
|  | **Selection** | **Comparability** | **Exposure** | **Total** |
| Bongsebandhu-Phubhakdi, 2008 | 4 | 0 | 3 | 7 |
| Marón, 2010 | 4 | 0 | 3 | 7 |
| Maneerattanasak, 2020 | 5 | 0 | 3 | 8 |
| Talukdar, 2021 | 4 | 0 | 3 | 7 |

**Table S4.** Main characteristics of included studies.

| Reference | Study design | Country | Study period | xCriteria | Dengue classification | | Age^a^ | Sex, males | Variable and criteria of obesity | Associated outcomes | Confirmatory diagnosis |
| --- | --- | --- | --- | --- | --- | --- | --- | --- | --- | --- | --- |
|  |  |  |  |  | **Dengue, n (%)** | **Severe dengue, n (%)** |  |  |  |  |  |
| Malavige 2006* | Cohort | Sri Lanka | April 24 to July 31 2004 | WHO 1997 | 18 (17.3%) DF 34 (39.5%) DHF grade I, 23 (26.7%) DHF grade II | 27 (31.4%) DHF grade III  2 (2.3%) DHF grade IV | 1 month to 12 years (mean 7.9 years, SD 2.9). | 41.3$ | Variable: BMI for age >90th centile | Neither BMI >90^th^ nor <5^th^ significantly different (P>0.05) in children with severe or mild disease, | Serological confirmation (IgG and IgM) |
| Bongsebandhu-Phubhakdi, 2008 | Cohort | Thailand | October 2004 to September 2006 | WHO 1997 | 46 (46.9%) DF | 52 (53.1%) DHF | 0–15 y  [children] | N/S | Variable: BW/age;  obesity: >110% | Nutritional status was not a risk factor for sever dengue | Serological confirmation; PCR |
| Marón, 2010 | Case-control | El Salvador | May to October 2004 | WHO 1997 | 74 (36.6%) Healthy controls;  66 (32.7%) DF | 62 (30.7%) DHF | 5–12 y;  Dengue cases: median (IQR) 101.1 (40.0) mo;  healthy controls: median (IQR) 100.4 (25.0) mo  [children] | Dengue cases: 43%;  healthy control: 39.1% | Variable: BAZ /HAZ/ WAZ;  overweight: BAZ > +2 | No significant differences in BW/age or BAZ between the dengue groups and the healthy control | Serological confirmation (IgM) |
| Maneerattanasak, 2020 | Cross-sectional | Thailand | January 2017 to December 2018 | WHO 1997 | 248 (43.4%) DF, 281 (49.1%) DHF grade I and II | 43 (7.5%) DSS (DHF grade III or IV) | 9.44±3.65 y (range: 1–14 y) [children] | 52.97% | Variable: BAZ/HAZ/WHZ;  overweight: BAZ > +2 SD (1–5 y), BAZ > +1 SD (5–14 y) according to WHO growth charts; WHZ > +1.5 SD according to Thai growth charts | Overweight was associated with increased risk of severe dengue | N/S |
| Talukdar, 2021 | Cohort | Thailand | March 2018 to February 2020 | Author-defined criteria | 349 (52.3%) Confirmed dengue without plasma leakage | 318 (47.7%) Confirmed dengue with plasma leakage | Median (IQR): 26 (20–37) y | 52.2% | Variable: BMI;  obesity: BMI ≥ 25.0 kg/m^2^ | Obese patients were more easily to develop plasma leakage (OR [95% CI]=1.478 [1.062–2.058], P=0.021) | Dengue NS1 test; serological confirmation (IgM);  (for all patient) rRT-PCR and micro-neutralization test |

^a^ mean ± SD unless otherwise indicated. *Malavige 2006 was not included in neither of our analysis due to cut-off point difference.

Abbreviations: DF, dengue fever; DHF, dengue hemorrhagic fever; DSS: dengue shock syndrome; BW/age, body weight-for-age; BMI, body mass index; BMI/age: BMI-for-age; BAZ, BMI-for-age Z-score; HAZ, height-for-age Z scores; WAZ, weight-for-age Z scores; WHZ, HAZ and weight-for-height Z scores; PCR, polymerase chain reaction; N/S, unspecified or unavailable.

1. Bongsebandhu-Phubhakdi C, Hemungkorn M, Thisyakorn U, Thisyakorn C: **Risk factors influencing severity in pediatric Dengue infection**. *Asian Biomedicine* 2008, **2**(5):409-413.

2. Maneerattanasak S, Suwanbamrung C: **Impact of Nutritional Status on the Severity of Dengue Infection Among Pediatric Patients in Southern Thailand**. *The Pediatric infectious disease journal* 2020, **39**(12):e410-e416.

3. Talukdar S, Thanachartwet V, Desakorn V, Chamnanchanunt S, Sahassananda D, Vangveeravong M, Kalayanarooj S, Wattanathum A: **Predictors of plasma leakage among dengue patients in Thailand: A plasma-leak score analysis**. *PLoS ONE* 2021, **16**(7 July 2021).

4. Marón GM, Clará AW, Diddle JW, Pleités EB, Miller L, Macdonald G, Adderson EE: **Association between nutritional status and severity of dengue infection in children in El Salvador**. *The American journal of tropical medicine and hygiene* 2010, **82**(2):324-329.

5. Tan VPK, Ngim CF, Lee EZ, Ramadas A, Pong LY, Ng JI, Hassan SS, Ng XY, Dhanoa A: **The association between obesity and dengue virus (DENV) infection in hospitalised patients**. *PLoS ONE [Electronic Resource]* 2018, **13**(7):e0200698.
