# Supplementary material for: Obesity as a clinical predictor for severe manifestation of dengue: a systematic review and meta-analysis
Source: BMC Infect Dis. 2023 Jul 31;23:502. doi: 10.1186/s12879-023-08481-9 (PMC10388491; doi:10.1186/s12879-023-08481-9)
Supplement: Supplementary file 5 — Supplementary Material 5 [file 12879_2023_8481_MOESM5_ESM.docx]

**Table S5.** Detailed scoring of Newcastle Ottawa-score.

| **Reference** | **Quality scores** | | | | | | | | |
| --- | --- | --- | --- | --- | --- | --- | --- | --- | --- |
|  | **Selection** | | | | **Comparability*** | | **Exposure** | | |
|  | (1) | (2) | (3) | (4) | (1a) | (1b) | (1) | (2) | (3) |
| Chuansumrit, 2000 | 1 | 1 | 1 | 1 | 0 | 0 | 1 | 1 | 1 |
| Basuki, 2003 | 1 | 1 | 1 | 1 | 0 | 0 | 1 | 1 | 1 |
| Kan, 2004 | 1 | 1 | 1 | 1 | 0 | 0 | 1 | 1 | 1 |
| Kalayanarooj, 2005 | 1 | 1 | 0 | 1 | 0 | 0 | 1 | 1 | 1 |
| Dewi, 2006 | 1 | 1 | 1 | 1 | 0 | 0 | 1 | 1 | 1 |
| Pichainarong, 2006 | 1 | 1 | 0 | 1 | 1 | 0 | 1 | 1 | 1 |
| Junia, 2007 | 1 | 1 | 0 | 1 | 0 | 0 | 1 | 1 | 1 |
| Tantracheewathorn, 2007 | 1 | 1 | 0 | 1 | 0 | 0 | 1 | 1 | 1 |
| Bongsebandhu-Phubhakdi, 2008 | 1 | 1 | 1 | 1 | 0 | 0 | 1 | 1 | 1 |
| Widagdo, 2008 | 1 | 1 | 1 | 2 | 0 | 0 | 2 | | 1 |
| Marón, 2010 | 1 | 1 | 1 | 1 | 0 | 0 | 1 | 1 | 1 |
| Widiyati, 2013 | 1 | 1 | 0 | 1 | 0 | 0 | 1 | 1 | 1 |
| Putra, 2014 | 1 | 1 | 0 | 1 | 0 | 0 | 1 | 1 | 1 |
| Lovera, 2016 | 1 | 1 | 1 | 1 | 0 | 0 | 1 | 1 | 1 |
| Tan, 2018 | 1 | 1 | 1 | 1 | 1 | 0 | 1 | 1 | 1 |
| Kurnia, 2019 | 0 | 1 | 0 | 1 | 0 | 0 | 1 | 1 | 1 |
| Maneerattanasak, 2020 | 1 | 1 | 1 | 2 | 0 | 0 | 2 | | 1 |
| Talukdar, 2021 | 1 | 1 | 1 | 1 | 0 | 0 | 1 | 1 | 1 |
| Te, 2022 | 1 | 0 | 1 | 2 | 0 | 0 | 2 | | 1 |
| *It is important to note that the absence of statistical significance between variables in the groups does not meet the criteria to be considered a star in the Newcastle-Ottawa score.. | | | | | | | | | |
